# The influence of sex-division, experience, and pacing strategy on performance in the 2020 CrossFit® Open

**DOI:** 10.3389/fspor.2024.1344036

**Published:** 2024-01-19

**Authors:** Gerald T. Mangine, Elisabeth K. Zeitz, Joshua D. Dexheimer, Ashley Hines, Brandon Lively, Brian M. Kliszczewicz

**Affiliations:** ^1^ Exercise Science, Kennesaw State University, Kennesaw, GA, United States; ^2^ Kinesiology, New Mexico State University, Las Cruces, NM, United States; ^3^ Health Sciences, Liberty University, Lynchburg, VA, United States

**Keywords:** high-intensity functional training, sport, competition, video analysis, repetition completion rate

## Abstract

To observe workout pacing strategies and determine which best predicted performance, this retrospective study analyzed recorded efforts from a random selection of 160 high-ranking (top 10,000) men and women (*n* = 80 each) in the 2020 CrossFit® Open (CFO). Video recordings submitted to the official competition leaderboard for all five tests were analyzed to quantify overall test completion rates (and tie-break time for test 5 only) and within-test repetition completion rate (repetitions × sec^−1^) for each exercise, as well as the quantity of failed repetitions, break strategy (count and duration), and transition times. Each variable was aggregated into first-half, last-half, and total-test averages, slopes, and coefficient of variation; except on test 5 (total-test only). Spearman's rank correlation coefficients were calculated between test completion rates, each test's respective pacing variables, competitor demographics (height and body mass) and CFO experience (i.e., past participation, consecutive competitions, and ranks). Stepwise regression using significantly (*p* < 0.05) correlated variables produced two prediction models for test performance (best predictor only and best overall model within 8 variables) in a validation group (50% of valid efforts) and then cross-validated against remaining athletes. When no between-group differences were seen, data were combined and used to create the final prediction models for test 1 (*r*^2^adj = 0.64–0.96, SEE = 0.4–1.2 repetitions × sec^−1^), test 2 (*r*^2^adj = 0.28–0.85, SEE = 2.0–4.5 repetitions × sec^−1^), test 3 (*r*^2^adj = 0.49–0.81, SEE = 1.1–1.7 repetitions × sec^−1^), test 4 (*r*^2^adj = 0.63–0.78, SEE = 0.6–0.9 repetitions × sec^−1^), and test 5 (rate: *r*^2^adj = 0.71–0.84, SEE = 1.2–1.6 repetitions × sec^−1^; tie-break time: *r*^2^adj = 0.06–0.62, SEE = 1.4–2.3 min). Across the five 2020 CFO tests, the data suggested that repetition pace, breaking strategy, and/or consistency in completing calisthenic-gymnastics components (when prescribed) was most predictive of performance. However, their influence was affected by the complexity of prescribed resistance training exercises and their relative loads. Athletes should prioritize calisthenic-gymnastics components but divert attention to more complex resistance training exercises when prescribed at higher relative intensity loads. Neither previous competition experience nor sex-division altered the hierarchal importance of these considerations.

## 1 Introduction

CrossFit® is a form of high-intensity functional training that aims to develop a wide array of fitness domains (10 are cited by the Level 1 Training Guide) through the constant variation of training stimuli (1). These workouts are typically considered to be “vigorous” based on reported elevations in heart rate (77%–95% of maximal heart rate) and blood lactate (≥13.3–18.9 mmol/L), last between a few to 30+ minutes in duration, and often challenge the trainee's strength and skill in performing any of the various (2–5+) exercise modalities that might appear (2–4). In addition to being a strategy for developing fitness, a wide variety of competitive sporting events feature CrossFit®-style workouts. The most popular, in terms of participation, is the CrossFit® Open (CFO) (5). The CFO is a global event that serves as the opening round of the CrossFit Games™ and consists of 3–6 tests of fitness through prescribed workouts that are announced individually over a span of 3–5 weeks via online broadcast (2, 3). Competitors who score well on these tests and earn an overall rank within a specified threshold (e.g., top 10%) advance to later rounds (i.e., quarterfinals, semifinals, Games™). Unlike future rounds, the CFO workouts are created with a restrictive list of potential exercises to make them more widely accessible (e.g., running and swimming have never been prescribed movements) with details of each test being unknown prior to their announcement. Competitors may utilize as many efforts as necessary to produce their best score within a 4-day window (2, 3). Although later rounds and other similar competitions (e.g., Wodapalooza, Granite Games, Rogue Invitational, etc.) might feature more advanced athletes, greater participation from competitors of all skill levels allow CFO performance to be a more generalizable metric of capability in this sport.

To ensure that performances can be distinguished and ranked, CFO tests have been structured to be scored in one (or in a combination) of three ways (2, 3). Competitors can be scored by weight lifted during a given task (e.g., clean and jerk, CNJ), or given a circuit of exercises to repeat for “as many repetitions as possible” (AMRAP, scored as repetitions completed) within a set time limit, or they are given a list of exercises to be completed as fast as possible within a time limit but scored as time-to-completion (TTC). This latter format might also be scored as repetitions completed if the competitor does not complete all tasks within a specified time limit. With the exception of workouts scored by weight lifted (only 6% of all CFO tests ever) (2, 3), the underlying instruction for all formats is maximizing workload density (i.e., more repetitions in less time) (6). Athletes can maximize workload density by completing exercise repetitions at a faster rate, avoiding failed repetitions, efficiently transitioning between exercises, or by limiting the number and duration of self-selected rest breaks. Of course, having command over these facets is not simply based on a conscious choice, but rather, the athlete's ability to manage fatigue over the duration of the test (7).

Managing fatigue is presumably dependent on the relative difficulty of the CFO test and could be hypothesized to be largely dependent on the athlete's physiological make up. Indeed, performance in different CrossFit®-style workouts have been related to a variety of physiological traits (8–15). However, none have consistently appeared to be most important across studied workouts. This may in part be explained by the various laboratory-based measures used across studies and their lack of specificity to the different CrossFit®-style workouts explored in these studies. It is also possible a trait's degree of relevance is modified by the competitor's training and competition experience (9, 14, 16). The more experienced competitor might better reconcile their own physiological capabilities with a correct perception of a test's demands to devise a more appropriate pacing strategy that best manages the accumulation of fatigue. In a recent study, pacing strategies were compared between men and women who ranked within the top 10% of the 2020 CFO and those within the top 10,000 (but not the top 10%) (17). In general, the top 10% (men and women) outpaced remaining competitors in approximately 60% of all exercises, more consistently transitioned between exercises, and were more consistent in their breaking strategy on half of tests. It was also noted that men exhibited a clear advantage in the gymnastics aspects of each test. While those data demonstrated sex and rank differences in pacing strategy, they did not reveal the meaningfulness of the observed differences. That is, knowing that top 10% competitors outperformed lower ranking athletes in nearly every facet of each test does not help to isolate which test aspects should receive the most attention to maximize performance.

The question of whether CFO test performance hinges on the pacing strategy employed for specific aspects about a test has only been addressed once in a pilot study on 2016 CFO recreational competitors (18). Being the first (and currently only) of its kind, that study's analysis predominantly pointed towards basic and intuitive concepts (e.g., average round completion rate) as the best predictors. The only specific strategy found was for test 4, where repetition completion rate for one of four programmed exercises [i.e., wall ball shots (WB)] was the most important determinant of performance. Otherwise, the take-home message suggested that recreational-level competitors should seek to maintain the fastest round completion rate possible to score best on 2016 CFO tests. Therefore, the purpose of this study was to expand on this concept using a large sample of high-ranking competitors and limiting assessed test elements to those that could be used to emphasize a specific strategic approach (e.g., repetition completion rate, failed repetitions, and breaking strategies for specific exercises only, transitions between specific exercises). A better understanding of which facets of pacing strategy are most important will not only assist athletes and coaches in devising an optimal approach but could also help to identify the most relevant (to the pacing aspect) physiological traits that might be considered for an efficient fitness testing battery. A secondary aim was to assess the influence of sex-division and competition experience on pacing strategy. Based on previous work (16), it was hypothesized that past CFO experiences (i.e., participation and success) would be influential of 2020 CFO test performance and modulate the importance of predictive pacing tactics.

## 2 Materials and methods

### 2.1 Experimental design

Demographic competition history data and video-recorded performances were collected from the official competition leaderboard (19) for a random sample of the top 10,000 men and women in the 2020 CFO (i.e., the most recent competition at the onset of this study). Each athlete's sex-division (men's or women's), height (cm), body mass (kg), and past competition performances (i.e., consecutive CFO appearances, skipped CFO competitions since first appearance, 2019 rank, and highest ever CFO rank before 2020) were recorded from the user profile linked to their rank placement on the leaderboard. Their video-recorded efforts were also accessed from the leaderboard via a camera icon placed next to their verified (by competition officials) score on each test (2, 3), and then analyzed using previously described methods (17). For the practical purposes of this paper, strategic pacing variables were selected based on their utility to coaches and athletes. A variable was considered useful if the athlete could intentionally focus on the tactic when completing the test (e.g., performing repetitions faster in a specific exercise) but not when it was synonymous with a test's final score (e.g., performing repetitions for all exercises at a faster rate would naturally lead to a faster TTC or total repetitions completed). Correlation analysis and stepwise regression were then used to determine the most influential tactics that resulted in the fastest pace (repetitions × minute^−1^) on each test, as well as the tie-break time on test 5. Since all data were pre-existing and publicly available, the University's Institutional Review Board classified data collected from this source for research purposes as exempt and did not require athletes to provide their informed consent (IRB #16-215).

### 2.2 Participants

The inclusion criteria for this study have been previously reported (17). All athletes ranked within the top 10,000 of their respective sex-division in the 2020 CFO and submitted video recordings of their best effort on all five 2020 CFO tests. Although a previous pilot study indicated a very strong predictive ability from individual pacing strategy variables across all five 2016 CFO tests (*r*^2^ = 0.89–0.99) (18), we opted for a more conservative *a priori* analysis approach. Using a moderate effect expectation for a linear multiple regression design (*f*^2^ = 0.15), sufficient power (*β* = 0.80–0.95) at standard alpha (*α* = 0.05) within the limit of eight predictive variables would be possible with 109–160 participants. Thus, 160 cases (80 men and 80 women) were randomly drawn from all athletes who met this study's initial inclusion criteria (men = 855, women = 416). Video submissions for these cases were screened for accuracy and completeness, and whenever a violation was observed, the entire case was replaced by a random selection from the pool of remaining eligible cases. Following the screening process, only data for a specific test was removed from the included 80 men and 80 women when an effort was observed to not meet programming standards during the video analysis process. This final process removed 10 cases (men = 4, women = 6) from test 1, five cases (men = 2, women = 3) from test 2, eight cases (men = 4, women = 4) from test 3, nine cases (men = 6, women = 3) from test 4, and seven cases (men = 6, women = 1) from test 5. Except for one woman's effort on test 3 (completed an incorrect test sequence), the reason for removal was miscounted repetitions. For correlation and regression analysis, the nominal variable of sex-division was encoded so that men were assigned a value of “0” and women “1”.

### 2.3 Competition test analysis

The 2020 CFO competition consisted of five tests released via live online broadcast once per week over five consecutive weeks beginning on 10 October 2019. Tests could be completed as initially prescribed (Rx) on the live broadcast for competitors in the men's and women's open divisions, or they could be completed with modified prescription if the athlete competed in one of the other competitive divisions (e.g., scaled, masters, teens, etc.). Because the modifications assigned to non-open divisions typically alter the assigned workload and overall test difficulty (2, 6), and less non-Rx competitors submit video recordings, this study only considered Rx efforts. Complete descriptions about the 2020 CFO's format, conditions for each test's release, and test and exercise standards are available elsewhere (2, 3). The requirements of each CFO test are briefly described, however, in Table 1. As Table 1 reveals, except for test 2, each test could be scored as TTC or as repetitions completed when a test's time limit expired. To quantify performance using a single outcome (dependent) variable, all official test scores were uniformly converted into a test completion rate (repetitions × minute^−1^), as previously recommended (6). A second outcome (dependent) variable, tie-break time (in minutes), was also used to describe test 5 performance, due to its enhanced importance towards ranking competitors who did not complete the entire test within 20 min.

**Table 1:** 2020 CFO test descriptions.

Test	Scoring	Prescription
1	TTC to complete 10 sets or repetitions completed in 15 min	Complete 10 sets of the following circuit:
		8 × Ground-to-overheads (G2OH; men: 95 lbs., women: 65 lbs.)
		10 × Bar-facing burpees (BFB)
2	Repetitions completed in 20 min	Complete “as many repetitions as possible” (AMRAP) of the following circuit:
		4 × Dumbbell thrusters (DBT; men: 50 lbs., women: 35 lbs.)
		6 × Toes-to-bar (TTB)
		24 × Double-unders (DU)
3	TTC for both circuits or repetitions completed in 9 min	Complete 21, 15, and 9 repetitions of circuit 1:
		Deadlifts (DL; men: 225 lbs., women: 155 lbs.)
		Handstand push-ups (HSPU)
		Then, complete 21, 15, and 9 repetitions of circuit 2:
		DL (men: 315 lbs., women: 205 lbs.)
		50′ Handstand walking (HSW)
4	TTC for both circuits or repetitions completed in 20 min	For circuit 1, alternate 30 × Box jumps (BJ; men: 24″ box height, women: 20″ box height) with:
		15 × CNJ (men: 95 lbs., women: 65 lbs.) → 15 × CNJ (men: 135 lbs., women: 85 lbs.) → 10 × CNJ (men: 185 lbs., women: 115 lbs.).
		For circuit 2, alternate 30 × Single-leg squats (SLSQ) with:
		10 × CNJ (men: 225 lbs., women: 145 lbs.) → 5 × CNJ (men: 275 lbs., women: 175 lbs.) → 5 × CNJ (men: 315 lbs., women: 205 lbs.)
5	TTC or repetitions completed in 20 min; tie-break time scored as TTC to complete 80 ROW and 120 WB	Complete the following prescription using any partitioning order:
		40 × Ring muscle-ups (RMU)
		80 × Rowing (ROW) calories
		120 × WB (men: 20 lbs. medicine ball to 10′ target, women: 14 lbs. medicine ball to 9′ target)

Video recordings for each workout were analyzed using previously described standardized methods to identify the start and end time for each test exercise, break, transition, and failed repetition (17). These data were entered into a spreadsheet (Microsoft Excel v. 365; Microsoft Corporation, Redmond, VA, USA) and used to calculate the pacing variables that would serve as this study's predicting (independent) variables. For tests 1, 3, and 4, the repetition completion rate (repetitions × s^−1^) for each exercise, the number of failed repetitions, average transition time between exercises, and the within-set breaking strategy (i.e., count, duration, and average) were quantified for each assigned set [i.e., sets 1–10 for test 1, and sets 1–6 (2 × 3-set circuits) for tests 3 and 4]. Then, the average, slope (variable per round), and coefficient of variation (CV; standard deviation divided by mean) were calculated for each independent variable over the first half, last half, and total duration of each test. The first half of test 1 was defined as sets 1–5, whereas sets 1–3 represented the first halves of tests 3 and 4. The remaining sets made up the last halves of each test. The same process was used for test 2 except that variables were quantified for each minute of the 20-minute workout, and the first half was defined as 0:00–9:59.

Variables were also quantified per minute for test 5, but because of the test's allotted partitioning freedom caused the halfway point to vary (in duration and work completed) for each athlete, calculations were limited to total test duration. Additionally, variables specific to test 5 only were also determined. These included the order of exercise completion and total sets used, and average repetitions per set for each exercise. The order of exercise completion assigned a numerical value (1, 2, or 3) to denote when an athlete completed all repetitions assigned to an exercise compared to the others. For example, a value of “1” was assigned to RMU if the athlete completed all 40 RMU repetitions before 80 ROW calories and/or 120 WB. Conversely, a value of “3” was assigned to the last exercise to be completed or if it remained unfinished after the 20-min time limit expired. Additionally, variables specific to ROW included the average calories per set, total rowing strokes, average rowing strokes per set, and average rowing calories per stroke. Non-specific exercise transitions [count, total duration per minute, and average transition duration (time in seconds divided by count)] were also included.

### 2.4 Statistical analysis

Results of the Shapiro-Wilks test indicated that several variables were not normally distributed, therefore Spearman's rank correlations were used to assess the relationships between all independent (i.e., demographic, experience, and test-specific pacing strategy variables) and dependent variables (i.e., test completion rate, and test 5 tie-break time). To identify the best predictors for each test, participants were randomly separated into validation and cross-validation groups using the random number function in Excel (v. 365, Microsoft Corp., Redmond, WA, USA). Due to differences in the number of athletes who met all requirements on each test, random group assignments were repeated for the analysis of each test. Validation groups were verified to possess no differences in demographic competitive history nor overall score on each test by independent-samples Mann–Whitney *U*-tests before proceeding to regression analysis.

Stepwise regression was performed in the validation group to predict repetition completion rate (repetitions × minute^−1^) on each test, and tie-break time (in minutes) on test 5, using significantly correlated demographic competition variables (except age) and test-specific pacing variables. From the list of significant models, two models of interest were identified: one that solely consisted of the single-best predictor and another that elicited the most precise estimate of test performance within the capacity of the present data set. Precision of the second model was based on variance explained by adjusted *r*-squared (*r*^2^_ADJ_) and the lowest standard error of the estimate (SEE) within eight predicting variables (i.e., approximately 20 participants per variable) (20). The suitability of each model was confirmed when its regression slope and intercept were significantly different from 0 to 1, respectively. Then, a new set of equations was developed by entering the same variables into a regression using data from the cross-validation group. When no significant differences existed between performance estimates produced by validation and cross-validation models according to Mann–Whitney *U*-tests, the data from both groups were pooled to generate the final prediction equations. The Shapiro–Wilks test was used to verify the normal distribution of residuals produced for each final model. All statistical analyses were performed using statistical Software (V. 29.0; SPSS Inc., Chicago, IL, USA) with a criterion alpha set at *p* ≤ 0.05. All data are reported as mean ± standard deviation (SD) or mean difference ± standard error (SE).

## 3 Results

Descriptive characteristics of the entire sample and validation groups for each variable are presented in Table 2. Except for the number of skipped CFO competitions since the athlete's initial appearance for test 1 validation groupings (*p* = 0.032), no significant differences for any measure were observed between validation groups.

**Table 2:** Validation group comparisons (mean ± SD).

	Test 1		Test 2		Test 3		Test 4		Test 5	
	V	CV	V	CV	V	CV	V	CV	V	CV
Age (years)	30.9 ± 5.6	31.6 ± 6.1	31.4 ± 6.4	30.4 ± 4.9	30.4 ± 4.6	31.7 ± 6.2	29.9 ± 5.1	31.8 ± 5.3	30.6 ± 4.7	31.6 ± 6.3
Height (cm)	171 ± 9	169 ± 9	170 ± 9	171 ± 8	170 ± 10	170 ± 8	170 ± 9	170 ± 9	170 ± 9	170 ± 8
Body mass (kg)	73.1 ± 13.7	71.5 ± 13.2	71.9 ± 14.4	72.8 ± 12.4	71.9 ± 14.4	72.0 ± 11.8	71.6 ± 14.1	72.5 ± 12.8	72.3 ± 13.2	72.1 ± 13.6
CFO participation										
Consecutive appearances (*n*)	3.7 ± 1.8	3.1 ± 1.6	3.4 ± 1.6	3.4 ± 2.0	3.0 ± 1.7	3.5 ± 1.8	3.1 ± 1.5	3.5 ± 1.9	3.5 ± 1.9	3.4 ± 1.6
Skipped competitions (*n*)	0.2 ± 0.5	0.2 ± 0.4*	0.2 ± 0.5	0.1 ± 0.4	0.2 ± 0.1	0.2 ± 0.5	0.2 ± 0.5	0.2 ± 0.4	0.2 ± 0.5	0.2 ± 0.4
Overall CFO ranking										
Highest rank ever (×1,000)	5.2 ± 6.3	9.4 ± 17.6	4.7 ± 5.5	9.6 ± 16.9	8.6 ± 16.0	6.4 ± 9.1	6.1 ± 6.8	6.5 ± 14.2	6.0 ± 8.8	5.9 ± 10.7
2019 rank (×1,000)	6.7 ± 9.7	12.5 ± 26.6	6.2 ± 9.5	12.4 ± 25.1	11.6 ± 25.1	6.9 ± 9.5	9.6 ± 21.4	7.7 ± 15.6	8.8 ± 20.7	7.2 ± 13.1
2020 rank (×1,000)	3.5 ± 2.8	3.8 ± 3.0	3.3 ± 2.7	3.9 ± 3.0	3.8 ± 3.0	3.7 ± 2.7	3.8 ± 3.0	3.3 ± 2.7	3.5 ± 2.7	3.2 ± 2.7
2020 percent rank (%)	86.2 ± 8.1	87.0 ± 6.3	88.1 ± 5.3	85.0 ± 9.1	86.8 ± 7.2	85.6 ± 8.2	86.7 ± 7.2	87.1 ± 7.0	86.7 ± 7.7	87.5 ± 6.3
2020 CFO test performance										
Test rank (×1,000)	6.3 ± 6.0	5.8 ± 5.4	4.0 ± 3.8	4.8 ± 4.8	6.2 ± 5.8	5.2 ± 4.4	6.0 ± 6.0	4.8 ± 5.7	4.0 ± 3.7	4.0 ± 3.7
Percent rank (%)	83.3 ± 15.2	82.9 ± 17.4	86.6 ± 14.1	86.9 ± 12.7	79.5 ± 20.8	84.8 ± 13.6	83.3 ± 17.9	87.2 ± 14.8	71.3 ± 30.8	72.7 ± 30.7
Time (min)	13 ± 1.6	13 ± 1.5	N/A		9.0 ± 0.1	9.0 ± 0.1	19.8 ± 0.6	19.7 ± 0.9	17.2 ± 2.9	17.3 ± 2.7
Repetitions completed (*n*)	179 ± 2	178 ± 6	706 ± 104	701 ± 98	120 ± 24	125 ± 20	211 ± 24	215 ± 20	232 ± 13	232 ± 12
Rate (repetitions × minute^−1^)	14.1 ± 2	13.9 ± 1.9	35.3 ± 5.2	35.0 ± 4.9	13.4 ± 2.7	14.0 ± 2.4	10.7 ± 1.4	11.0 ± 1.5	14.0 ± 3.2	13.9 ± 2.9
Tie-break time (min)	N/A		N/A		N/A		N/A		15.2 ± 2.2	15.6 ± 2.3

V, validation group; CV, cross-validation group.

* Significantly (*p* < 0.05) different between validation groups.

For demographic competition history variables, significant (*p* < 0.05) negative relationships were observed between repetition completion rate on all five tests and sex-division and overall ranking in previous CFO competitions (highest rank earned ever and 2019 rank). These suggests faster repetition completion rates were seen in men and competitors who ranked well in past competitions. Readers interested in reviewing specific comparisons between men and women can find those here (17). Meanwhile, the significant (*p* < 0.05) positive relationships noted for repetition completion rate with body mass and height imply that taller and heavier competitors performed repetitions at a faster rate on each test. The number of consecutive CFO appearances since the athlete's initial CFO appearance was only related to test 3 and test 4 completion rate, with more consecutive appearances being associated with a faster repetition completion rate. The number of CFO competitions skipped since the athlete's first appearance was not related to performance on any test. None of these variables were related to test 5 tie-break time. Relationships between demographic competition history variables and test performance are presented in Table 3.

**Table 3:** Relationships between demographic competition history and test performance.

	Test 1	Test 2	Test 3	Test 4	Test 5	
					Rate	Tie- break
Sex-division	−0.32*	−0.39*	−0.26*	−0.18*	−0.78*	0.14
Height	0.30*	0.35*	0.22*	0.24*	0.69*	−0.12
Body mass	0.40*	0.41*	0.39*	0.36*	0.75*	−0.16
CFO participation						
Consecutive appearances	0.14	0.13	0.17*	0.24*	0.08	0.07
Skipped competitions	0.01	−0.05	0.02	0.06	0.01	0.03
Overall CFO ranking						
Highest rank ever	−0.68*	−0.67*	−0.73*	−0.69*	−0.48*	0.13
2019 rank	−0.67*	−0.67*	−0.78*	−0.71*	−0.48*	0.12

* Significantly (*p* < 0.05) related to test performance.

**Figure 1:**
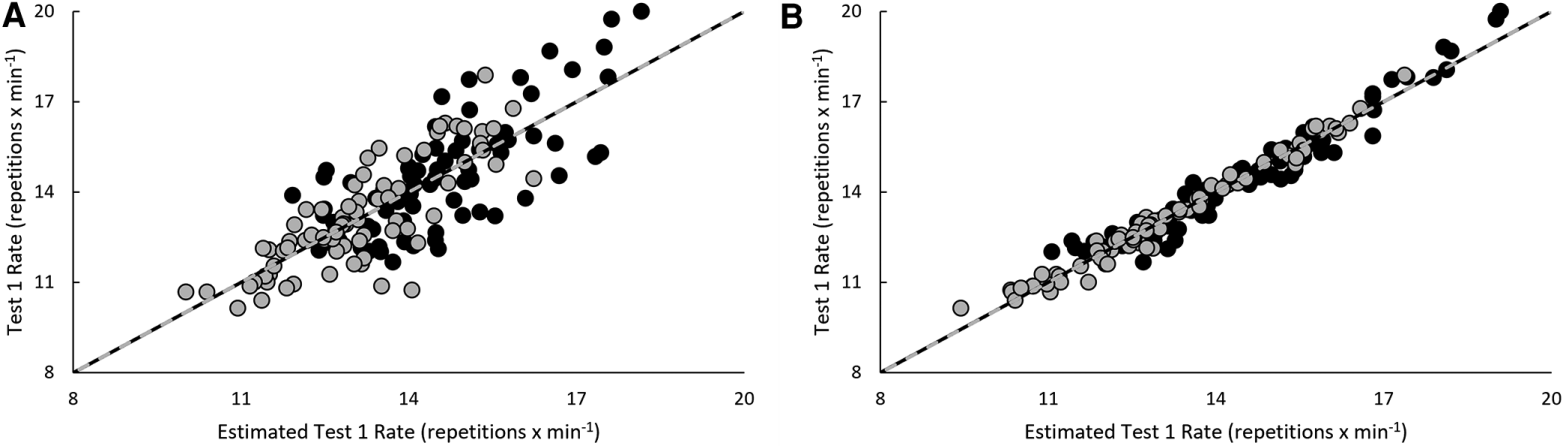
Regression line comparison between the derived equations and observed test 1 rate for the (**A**) best predictor and (**B**) best estimate. *Gray dashed line = line of identity (slope = 1); black solid line = line of best fit from linear regression; Black circles = Men; Grey circles = Women.

**Figure 2:**
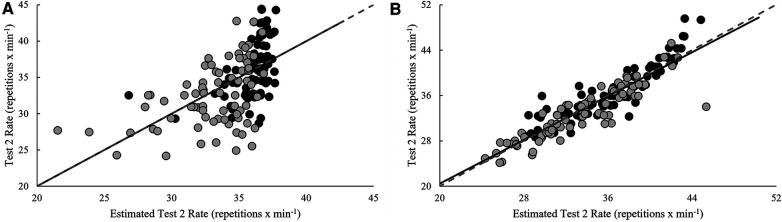
Regression line comparison between the derived equations and observed test 2 rate for the (**A**) best predictor and (**B**) best estimate. *Gray dashed line = line of identity (slope = 1); black solid line = line of best fit from linear regression; Black circles = Men; Grey circles = Women.

**Figure 3:**
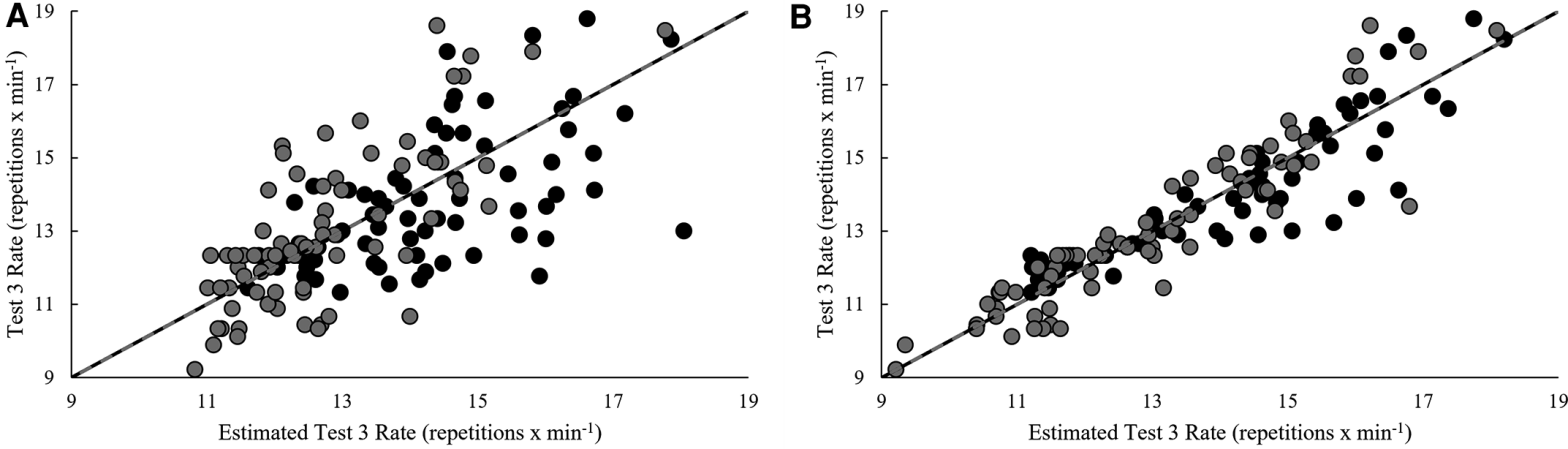
Regression line comparison between the derived equations and observed test 3 rate for the (**A**) best predictor and (**B**) best estimate. *Gray dashed line = line of identity (slope = 1); black solid line = line of best fit from linear regression; Black circles = Men; Grey circles = Women.

**Figure 4:**
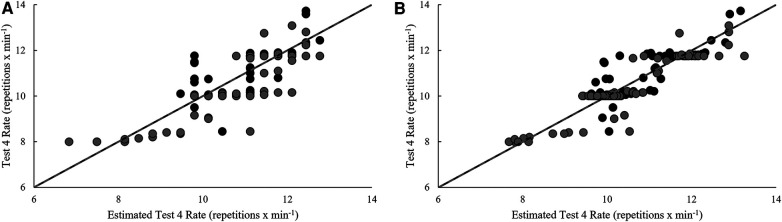
Regression line comparison between the derived equations and observed test 4 rate for the (**A**) best predictor and (**B**) best estimate. *Gray dashed line = line of identity (slope = 1); black solid line = line of best fit from linear regression; Black circles = Men; Grey circles = Women.

**Figure 5:**
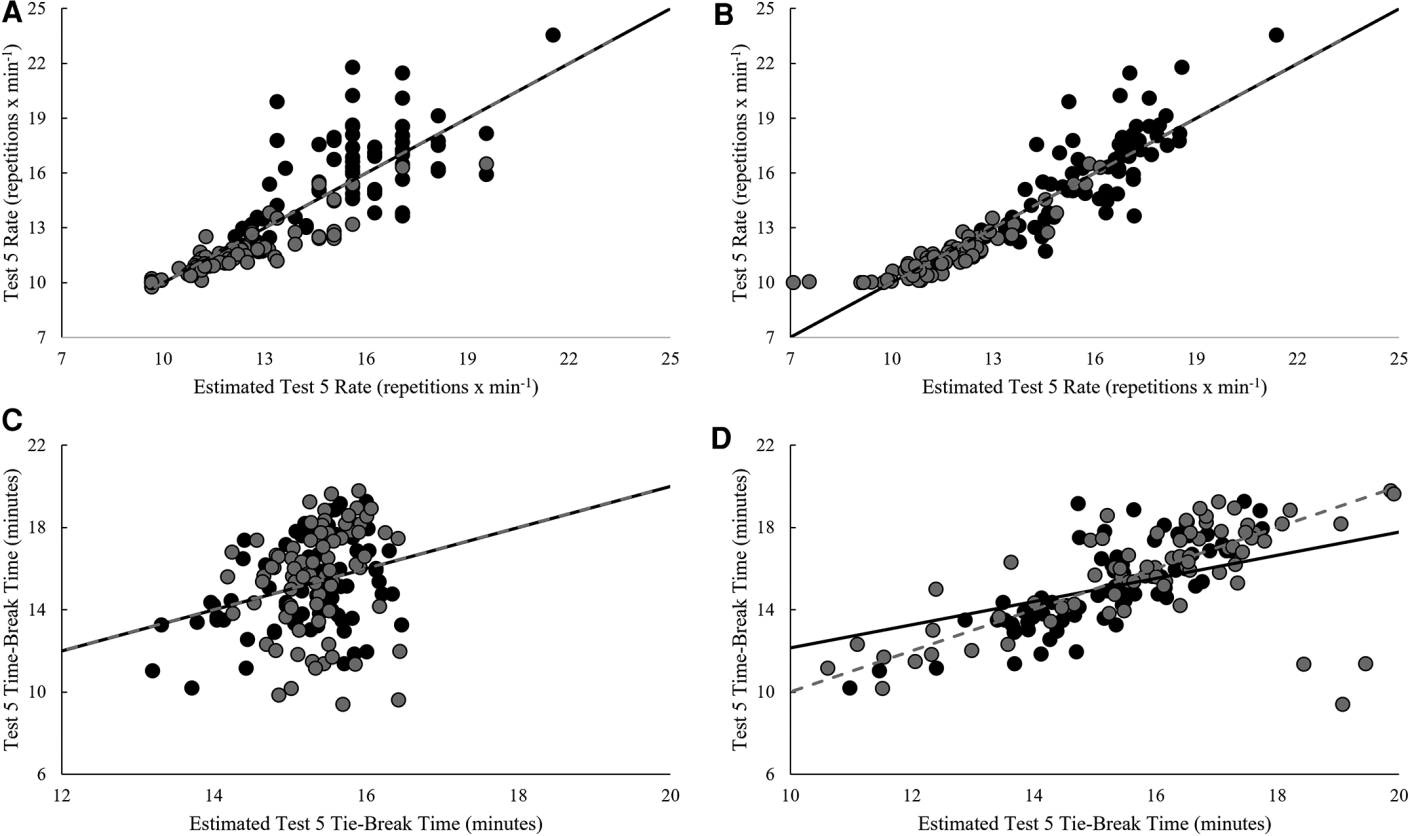
Regression line comparison between the derived equations and observed (**A**) test 5 rate for the best predictor, (**B**) test 5 rate for the best estimate, (**C**) test 5 tie-break time for the best predictor, and (**D**) test 5 tie-break time for the best estimate. *Gray dashed line = line of identity (slope = 1); black solid line = line of best fit from linear regression; Black circles = Men; Grey circles = Women.

Model estimates derived from validation and cross-validation group data for each test, as well as final model estimates, using the single-best and best combination of variables are presented in Table 4. Relationships between pacing strategy variables and test performance are presented in Tables 5–7, while models using the best predictor and variables that produced the most precise estimates of performance are illustrated in Figures 1–5.

**Table 4:** Cross-validated prediction equations of 2020 CFO perfromance using the single-best predictor and best collection of pacing strategy variables to produce the best overall overall model.

	Best predictor			Best model		
Test 1 rate (repetitions × min^−1^)						
Validation	= 2.25 + (49.23 × average BFB rate [reps × sec-1]	14.0 ± 1.6	0.870	= 3.80 + (36.02 × average BFB rate [reps × sec-1]) + (9.92 × average last-half G2OH rate [reps × sec-1]) − (0.21 × average transitions time to G2OH [sec]	13.9 ± 2.0	0.797
Cross-validation	= 2.53 + (48.12 × average BFB rate [reps × sec-1]	13.8 ± 1.6		= 3.93 + (35.89 × average BFB rate [reps × sec-1]) + (9.53 × average last-half G2OH rate [reps × sec-1]) − (0.20 × average transitions time to G2OH [sec]	13.8 ± 1.9	
Total	= 2.40 + (48.42 × average BFB rate [reps × sec-1]			= 3.64 + (36.84 × average BFB rate [reps × sec-1]) + (9.55 × average last-half G2OH rate [reps × sec-1]) − (0.20 × average transitions time to G2OH [sec]		
Test 2 rate (repetitions × min^−1^)						
Validation	= 40.72 − (43.75 × TTB rate CV [in decimals]	35.3 ± 3.6	0.239	= 53.98 − (22.07 × TTB rate CV [in decimals]) − (0.70 × average transition to DBT [sec]) − (5.96 × average first-half TTB breaks [n]) − (1.15 × average first-half transition to DU [sec]) − 21.78 × DU rate CV [in decimals])	35.3 ± 5.0	0.918
Cross-validation	= 38.10 − (35.86 × TTB rate CV [in decimals]	34.6 ± 2.4		= 53.10 − (17.29 × TTB rate CV [in decimals]) − (0.61 × average transition to DBT [sec]) − (3.00 × average first-half TTB breaks [n]) − (1.24 × average first-half transition to DU [sec]) − 20.01 × DU rate CV [in decimals])	35.2 ± 4.6	
Total	= 38.91 − (39.49 × TTB rate CV [in decimals]			= 53.45 − (21.01 × TTB rate CV [in decimals]) − (0.72 × average transition to DBT [sec]) − (4.18 × average first-half TTB breaks [n]) − (1.10 × average first-half transition to DU [sec]) − 18.84 × DU rate CV [in decimals])		
Test 3 rate (repetitions × min^−1^)						
Validation	= 8.62 + (14.67 × average first-half HSPU rate [reps × sec-1]	13.6 ± 1.5	0.756	= 8.42 + (7.13 × average first-half HSPU rate [reps × sec-1]) + (9.32 × average last-half DL rate [reps × sec-1]) + (8.50 × average last-half HSW rate [reps × sec-1]	35.3 ± 3.6	0.239
Cross-validation	= 10.18 + (10.58 × average first-half HSPU rate [reps × sec-1]	13.7 ± 1.5		= 9.12 + (4.56 × average first-half HSPU rate [reps × sec-1]) + (10.40 × average last-half DL rate [reps × sec-1]) + (8.44 × average last-half HSW rate [reps × sec-1]	34.6 ± 2.4	
Total	= 9.61 + (11.93 × average first-half HSPU rate [reps × sec-1]			= 8.63 + (5.74 × average first-half HSPU rate [reps × sec-1]) + (11.27 × average last-half DL rate [reps × sec-1]) + (7.56 × average last-half HSW rate [reps × sec-1]		
Test 4 rate (repetitions × min^−1^)						
Validation	= 6.57 + (1.06 × average last-half CNJ break count)	10.5 ± 1.17	0.239	= 7.77 + (0.99 × average last-half CNJ break count) − [3.46 × slope in CNJ failed repetitios (reps × round-1)] + [0.66 × CV of last-half CNJ rate (reps × sec-1)] − [0.01 × average last-half CNJ break time (sec)]	10.6 ± 1.4	0.674
Cross-validation	= 7.02 + (0.94 × average last-half CNJ break count)	10.8 ± 1.02		= 7.84 + (1.10 × average last-half CNJ break count) − (2.98 × slope in CNJ failed repetitios [reps × round-1]) + (0.61 × CV of last-half CNJ rate [reps × sec-1]) − (0.02 × average last-half CNJ break time [sec])	10.7 ± 1.1	
Total	= 6.84 + (0.99 × average last-half CNJ break count)			= 7.89 + (1.04 × average last-half CNJ break count) − (3.04 × slope in CNJ failed repetitions [reps × round-1]) + (0.61 × CV of last-half CNJ rate [reps × sec-1]) − (0.01 × average last-half CNJ break time [sec])		
Test 5 rate (repetitions × min^−1^)						
Validation	= 9.29 + (1.65 × average RMU repetitions per set)	13.6 ± 2.8	0.608	= 7.84 + (0.75 × average RMU repetitions per set) − (0.46 × RMU order of completion [1,2,3]) − (10.99 × Slope of transitions [count × min-1]) + (0.08 × body mass [kg]) − (0.19 × average transition time [sec]) + (1.14 × average transition count)	13.2 ± 3.8	0.565
Cross-validation	= 9.75 + (1.42 × average RMU repetitions per set)	13.7 ± 2.5		= 11.53 + (0.91 × average RMU repetitions per set) − (0.68 × RMU order of completion [1,2,3]) − (5.94 × Slope of transitions [count × min-1]) + (0.03 × body mass [kg]) − (0.13 × average transition time [sec]) + (0.59 × average transition count)	13.7 ± 2.7	
Total	= 9.66 + (1.48 × average RMU repetitions per set)			= 10.09 + (0.77 × average RMU repetitions per set) − (0.84 × RMU order of completion [1,2,3]) − (4.83 × Slope of transitions [count × min-1]) + (0.05 × body mass [kg]) − (0.08 × average transition time [sec]) + (0.44 × average transition count)		
Test 5 tie-break time (minutes)						
Validation	= 16.59 + (17.19 × slope of transitions [count × min-1])	15.5 ± 1.2	0.253	= 5.95 + (10.27 × slope of transitions [count × min-1]) + (0.30 × average transition time [sec]) + (0.04 × rowing strokes) − (0.21 × average RMU break time [sec]) + (2.47 × average RMU break count)	15.9 ± 2.8	0.729
Cross-validation	= 15.98 + (11.42 × slope of transitions [count × min-1])	15.4 ± 0.7		= 7.45 + (9.08 × slope of transitions [count × min-1]) + (0.25 × average transition time [sec]) + (0.03 × rowing strokes) − (0.28 × average RMU break time [sec]) + (4.26 × average RMU break count)	15.5 ± 1.9	
Total	= 15.82 + (9.26 × slope of transitions [count × min-1])			= 8.16 + (9.12 × slope of transitions [count × min-1]) + (0.21 × average transition time [sec]) + (0.03 × rowing strokes) − (0.26 × average RMU break time [sec]) + (3.67 × average RMU break count)		

**Table 5:** Relationships between 2020 CFO tests 1 and 2 performance and select pacing strategy variables.

	Average			Slope			Coefficient of variation		
	First half	Last half	Total	First half	Last half	Total	First half	Last half	Total
Test 1									
Ground-to-Overhead									
Rate (repetitions × round^−1^)	0.61*	0.60*	0.63*	0.18*	0.01	0.26*	−0.32*	−0.35*	−0.43*
Failed repetitions (*n*)	−0.04	0.02	−0.01						
Breaks (*n*)	−0.29*	−0.37*	−0.39*	−0.30*	−0.11	−0.29*	−0.29*	−0.23*	−0.27*
Total break time (s)	−0.29*	−0.42*	−0.43*	−0.32*	0.01	−0.41*	−0.31*	−0.26*	−0.28*
Average break time (s)	−0.30*	−0.43*	−0.44*	−0.32*	0.04	−0.41*	−0.31*	−0.26*	−0.28*
Bar-facing burpees									
Rate (repetitions × round^−1^)	0.75*	0.68*	0.77*	−0.01	0.13	0.05	−0.17*	0.03	−0.09
Failed repetitions (*n*)	−0.22*	−0.07	−0.19*						
Breaks (*n*)	−0.19*	0.01	−0.05	−0.11	0.08	0.04	−0.18*	0.04	−0.02
Total break time (s)	−0.15	0.01	−0.03	−0.16*	0.12	0.03	−0.15	0.04	0.01
Average break time (s)	−0.16	0.01	−0.04	−0.16*	0.08	0.03	−0.15	0.04	0.01
Transition time (s)									
To bar-facing burpees	−0.50*	−0.37*	−0.44*	−0.29*	0.35*	−0.01	−0.01	−0.03	0.06
To ground-to-overheads	−0.57*	−0.50*	−0.57*	−0.41*	0.35*	−0.15	−0.20*	−0.16*	−0.11
Test 2									
Dumbbell Thrusters									
Rate (repetitions × sec^−1^)	0.50*	0.49*	0.50*	−0.05	0.13	0.07	−0.38*	−0.35*	−0.46*
Failed repetitions (*n*)	0.01	0.03	0.03	0.01	0.03	0.03	0.01	0.03	0.03
Breaks (*n*)	0.01	0.01	0.01	0.01	0.09	0.01	0.01	0.01	0.01
Total break time (s)	0.01	0.01	0.01	0.01	0.09	0.01	0.01	0.01	0.01
Average break time (s)	0.01	0.01	0.01	0.01	0.09	0.01	0.01	0.01	0.01
Toes-to-bar									
Rate (repetitions × sec^−1^)	0.29*	0.44*	0.40*	0.03	0.22*	0.32*	−0.46*	−0.50*	−0.61*
Failed repetitions (*n*)	−0.15	−0.11	−0.13	−0.01	−0.09	0.01	−0.15	−0.11	−0.13
Breaks (*n*)	−0.27*	−0.23*	−0.25*	0.01	−0.06	−0.03	−0.24*	−0.16*	−0.17*
Total break time (s)	−0.27*	−0.22*	−0.24*	−0.19*	0.01	−0.12	−0.27*	−0.13	−0.15
Average break time (s)	−0.27*	−0.24*	−0.26*	−0.19*	−0.02	−0.14	−0.27*	−0.16*	−0.18*
Double-unders									
Rate (repetitions × sec^−1^)	0.41*	0.40*	0.43*	−0.14	−0.01	−0.07	−0.48*	−0.49*	−0.57*
Failed repetitions (*n*)	−0.20*	−0.20*	−0.24*	0.19*	−0.06	0.06	−0.05	−0.09	−0.10
Breaks (*n*)	0.07	−0.02	0.01	−0.04	−0.02	−0.08	0.15	0.12	0.10
Total break time (s)	0.01	−0.10	−0.08	−0.11	0.01	−0.12	0.14	0.10	0.07
Average break time (s)	0.01	−0.12	−0.09	−0.1	−0.02	−0.15	0.14	0.10	0.07
Average transition time									
To toes-to-bar (s)	−0.63*	−0.55*	−0.63*	−0.39*	0.03	−0.18*	−0.59*	−0.47*	−0.58*
To double-unders (s)	−0.61*	−0.53*	−0.59*	−0.06	0.23*	0.05	−0.39*	−0.49*	−0.44*
To dumbbell thrusters (s)	−0.70*	−0.62*	−0.69*	−0.36*	0.29*	−0.06	−0.54*	−0.55*	−0.58*

* Significantly (*p* < 0.05) related to test performance.

**Table 6:** Relationships between 2020 CFO tests 3 and 4 performance and select pacing strategy variables.

	Average			Slope			Coefficient of variation		
	First half	Last Half	Total	First half	Last half	Total	First half	Last half	Total
Test 3									
Deadlifts									
Rate (repetitions × round^−1^)	0.53*	0.62*	0.36*	−0.05	−0.34*	0.09	−0.21*	−0.40*	0.01
Failed repetitions (*n*)	−0.09	−0.12	−0.15						
Breaks (*n*)	−0.15	−0.28*	−0.05	−0.01	0.47*	−0.22*	0.12	−0.40*	−0.18*
Total break time (s)	−0.32*	−0.33*	−0.11	0.03	0.75*	−0.27*	0.04	−0.57*	−0.09
Average break time (s)	−0.28*	−0.06	−0.06	−0.06	0.51*	0.01	0.06	−0.37*	0.05
Handstand push-ups/walks									
Rate (repetitions × round^−1^)	0.71*	0.71*	0.62*	−0.37*	−0.22	−0.31*	−0.04	−0.25	0.13
Failed repetitions (*n*)	−0.34*	0.11	−0.29*						
Breaks (*n*)	−0.6*	−0.28*	−0.51*	0.31*	0.49*	0.41*	−0.18*	−0.42*	−0.30*
Total break time (s)	−0.67*	−0.22*	−0.62*	0.35*	0.69*	0.41*	−0.25*	−0.73*	−0.30*
Average break time (s)	−0.52*	0.15	−0.48*	0.14	0.71*	0.34*	−0.10	−0.75*	−0.42*
Average transition time (s)									
To handstand push-ups/walks	−0.60*	−0.14	−0.47*	−0.28*	0.58*	−0.22*	−0.12	−0.30	−0.17*
To deadlifts	−0.20*	0.17	−0.19*	0.16*	0.78*	−0.23*	0.27*	−0.86*	−0.24*
Test 4									
Box jumps/Single-leg squats									
Rate (repetitions × round^−1^)	0.40*	0.18*	0.39*	0.06	−0.08	0.04	−0.16	0.76*	−0.10
Failed repetitions (*n*)	0.04	−0.07	−0.03	−0.04	0.24*	−0.07	0.03	−0.05	−0.03
Breaks (*n*)	−0.07	0.03	0.01	−0.06	0.40*	0.18*	−0.05	−0.04	−0.08
Total break time (s)	−0.07	0.01	−0.03	−0.06	0.40*	0.12	−0.06	−0.01	−0.06
Average break time (s)	−0.07	0.04	0.01	−0.05	0.39*	0.23*	−0.06	−0.02	−0.06
Clean-and-jerks									
Rate (repetitions × round^−1^)	0.63*	0.81*	0.41*	−0.06	−0.22*	0.32*	−0.47*	0.74*	−0.27*
Failed repetitions (*n*)	0.01	−0.29*	−0.29*	0.01	0.63*	−0.04	0.01	−0.07	−0.07
Breaks (*n*)	−0.10	0.83*	0.26*	−0.29*	0.28*	0.37*	−0.08	−0.82*	−0.63*
Total break time (s)	−0.47*	−0.01	−0.26*	−0.65*	0.79*	0.67*	−0.12	−0.77*	−0.66*
Average break time (s)	−0.36*	0.28*	0.12	−0.35*	0.83*	0.61*	−0.06	−0.64*	−0.34*
Average transition time (s)									
To clean-and-jerk	−0.54*	−0.43*	−0.39*	−0.46*	0.15	−0.36*	−0.30*	0.78*	0.09
To box jumps/single-leg squats	−0.46*	−0.30*	−0.40*	−0.15	−0.49*	0.07	−0.06	0.67*	−0.10

* Significantly (*p* < 0.05) related to test performance.

**Table 7:** Relationships between 2020 CFO test 5 performance and select pacing strategy variables.

	Rate (repetitions × min^−1^)			Tie-break time (s)		
	Ring muscle-ups	Rowing	Wall ball shots	Ring muscle-ups	Rowing	Wall ball shots
Order of completion	−0.77*	0.50*	0.34*	0.21*	−0.29*	0.07
Total sets	−0.30*	−0.19*	−0.03	0.50*	0.31*	0.31*
Repetitions/calories per set	0.91*	0.19*	0.03	−0.07	−0.31*	−0.31*
Total rowing strokes	-	−0.49*	-	-	0.40*	-
Rowing strokes per set	-	0.05	-	-	−0.18*	-
Rowing calories per stroke	-	0.49*	-	-	−0.40*	-
Repetition completion rate	0.90*	0.58*	0.04	−0.05	−0.42*	0.01
Failed repetitions	−0.43*		−0.03	0.13		0.06
Per minute	Average	Slope	Coefficient of variation	Average	Slope	Coefficient of variation
Ring muscle-ups						
Repetition completion rate	0.89*	0.17*	−0.61*	−0.04	−0.07	0.22*
Break count	−0.77*	−0.50*	−0.26*	−0.02	−0.11	0.12
Break time	−0.80*	−0.54*	−0.26*	−0.05	−0.16	0.12
Rowing						
Strokes	0.21*	0.29*	0.20*	−0.04	−0.12	−0.06
Calories per stroke	0.22*	0.28*	0.20*	−0.04	−0.12	−0.06
Break count	−0.07	0.10	−0.07	0.09	−0.06	0.09
Break time	−0.07	0.10	−0.07	0.09	0.01	0.09
Wall ball shots						
Repetition completion rate	−0.02	−0.07	−0.10	0.01	−0.08	0.16
Break count	−0.35*	−0.18*	−0.27*	0.04	0.02	0.15
Break time	−0.34*	−0.25*	−0.28*	0.06	0.01	0.15
Transitions						
Time devoted	−0.32*	−0.33*	−0.32*	0.20*	−0.41*	−0.22*
Count	0.48*	−0.11	−0.34*	0.22*	0.28*	−0.33*
Average	0.38*	−0.01	−0.35*	−0.19*	0.35*	0.01

* Significantly (*p* < 0.05) related to test performance.

### 3.1 Test 1

Test 1 completion rate was positively related (*p* < 0.05) to average G2OH and BFB repetition completion rates across the entire test, the slope of G2OH rate in the first five rounds, and the slope of average transitions during the last five rounds. Negative relationships (*p* < 0.05) were seen with CVs of BFB rate over 10 rounds, the averages of and CVs of BFB breaks (count, total duration, and average duration) over the entire test, and their slope during the first five rounds. Average transition times to each exercise over 10 rounds, their slope in the first five rounds, and CVs when transitioning to G2OH over 10 rounds were also negatively related to test 1 completion rate (*p* < 0.05). Of these, average burpee rate over all ten rounds was the best predictor of performance (*r*^2^_ADJ_ = 0.64, SEE = 1.19 repetitions **× **min^−1^, *p* < 0.001). Variance explained was improved by 32.3% with the inclusion of average G2OH rate over the last five rounds and average transition time to G2OH in the best model (*r*^2^_ADJ_ = 0.96, SEE = 0.39 repetitions × min^−1^, *p* < 0.001).

### 3.2 Test 2

Test 2 completion rate was positively related (*p* < 0.05) to the repetition completion rate of all three exercises over the duration of the workout, the slope in TTB rate (last-half and overall), failed DU repetitions (first-half), and last-half transition time to DU and DBT. Throughout the entire 20-minute test, negative relationships (*p* < 0.05) were seen with the CVs of all three exercises, average TTB breaks (count, total and average duration), the CVs of TTB break count and average break duration, and average failed DU repetitions. Within the first 10 min only, negative relationships (*p* < 0.05) were seen the slope of TTB breaks (total and average duration) and the CV of total TTB break duration. Of these, test 2 performance was best predicted by the CV for TTB rate over 20 min (*r*^2^_ADJ_ = 0.28, SEE = 4.46 repetitions **× **min^−1^, *p* < 0.001). Variance explained was improved by 56.7% with the inclusion of average transition time to DBT, first-half TTB break count, first-half transition time to DU, and the CV of DU rate in the best model (*r*^2^_ADJ_ = 0.85, SEE = 2.00 repetitions **× **min^−1^, *p* < 0.001).

### 3.3 Test 3

Completion rate of test 3's six rounds was positively related (*p* < 0.05) to the repetition completion rate of each exercise over all six rounds, the slopes of HSPU/HSW breaks (count, total and average duration), and the CVs of transitions to DL and HSPU/HSW. Positive relationships (*p* < 0.05) were also seen with the slopes of DL breaks (count, total and average duration) over the last three rounds. Negative relationships (*p* < 0.05) were observed with the slope of HSPU rate, average HSPU/HSW failed repetitions and breaks (count, total and average duration), CVs with HSPU/HSW breaks (count, total and average duration), and with the average and slopes of transition times to each exercise over the entire test. The CVs of DL breaks (count, total and average duration) over the last three rounds were negatively related to test 3 completion rate (*p* < 0.05), while relationships observed with the slopes of DL breaks over the last three rounds and overall were not in agreement. Test 3 performance was best predicted by average HSPU rate (*r*^2^_ADJ_ = 0.49, SEE = 1.73 repetitions × min^−1^, *p* < 0.001). Variance explained was improved by 31.8% with the inclusion of average DL and HSW rates over the last three rounds in the best model (*r*^2^_ADJ_ = 0.81, SEE = 1.06 repetitions × min^−1^, *p* < 0.001).

### 3.4 Test 4

Positive relationships (*p* < 0.05) were noted over six rounds of test 4 and repetition completion rate of both exercises, the slope of BJ-SLSQ breaks (count and average duration), average CNJ break count, and the slopes of CNJ failed repetitions and breaks (count, total and average duration). Positive relationships (*p* < 0.05) were also noted for last-half CV of SLSQ rate, slope of SLSQ breaks (count and total duration), the CV of CNJ rate, average CNJ break duration, and CV of transitions. Of these, test 4 performance was best predicted by average last-half CNJ break count (*r*^2^_ADJ_ = 0.63, SEE = 0.85 repetitions **× **min^−1^, *p* < 0.001). Variance explained was improved by 15.9% with the inclusion of slope in failed CNJ repetitions, CV of last-half CNJ rate, and average last-half CNJ break time in the best model (*r*^2^_ADJ_ = 0.78, SEE = 0.65 repetitions **× **min^−1^, *p* < 0.001).

### 3.5 Test 5

Test 5 was different from other tests because athletes were free to partition the required work in any way. Further, because 42.5% (*n* = 65) of athletes did not complete all 40 RMU repetitions, the tie-break score (i.e., time to complete 80 rowing calories and 120 WB) was significantly related to overall repetition completion rate (*r* = −0.19, *p* = 0.017) and acted as the final score for those athletes who could not complete a single RMU repetition (*n* = 9).

Test 5 completion rate was positively related (*p* < 0.05) to overall strategy for order of completion (ROW and WB, i.e., completing these after RMU was associated with a faster repetition completion rate), RMU performance (repetitions per set and rate), and ROW performance (calories per set, calories per stroke, and calories per stroke per set). Positive relationships (*p* < 0.05) were also noted for per minute strategy with RMU rate (average and slope), ROW strokes (average, slope, and CV), ROW calories per stroke (average, slope, and CV), and average transitions (count and average duration). Completion rate was negatively related (*p* < 0.05) to overall strategy for order of completion (RMU), RMU performance (total sets and failed repetitions), and total ROW strokes. Negative relationships (*p* < 0.05) were also noted for per minute strategy with the CV of RMU rate, RMU break count and time (average, slope, and CV), WB break count and time (average, slope, and CV), time devoted to transitions (average, slope, and CV), and the CVs for transition counts and average durations. Of these, the best predictor of test 5 repetition completion rate was average RMU repetitions per set (*r*^2^_ADJ_ = 0.71, SEE = 1.62 repetitions **× **min^−1^, *p* < 0.001). Variance explained was improved by 12.7% with the inclusion of the order of RMU repetition completion (i.e., 1st, 2nd, or 3rd), the slope and average number of transitions over workout duration, the average duration of each transition, and the athlete's reported body mass in the best model (*r*^2^_ADJ_ = 0.84, SEE = 1.21 repetitions **× **min^−1^, *p* < 0.001).

Test 5 tie-break time was positively related (*p* < 0.05) to overall order of completion (RMU), total sets (RMU, ROW, and WB), and total rowing strokes. Positive relationships (*p* < 0.05) were also noted for per minute strategy with the CV of RMU rate, average transitions (count and total time devoted), and the slope of transitions (count and average duration). Test 5 tie-break time was negatively related (*p* < 0.05) to overall strategy for ROW (order of completion, calories per set, strokes per set, calories per stroke, and calories per stroke per set) and WB repetitions per set. Negative relationships (*p* < 0.05) were also noted for per minute strategy with the CV of transition count, total time devoted to transitions (slope and CV), and average transition duration. Of these, tie-break time itself was best predicted by the slope of transition count over the workout duration (*r*^2^_ADJ_ = 0.06, SEE = 2.27 repetitions **× **min^−1^, *p* < 0.001). Variance explained was improved by 55.7% with the inclusion of average transition time, total rowing strokes, and average RMU break count and break duration (*r*^2^_ADJ_ = 0.62, SEE = 1.38 repetitions × min^−1^, *p* < 0.001).

## 4 Discussion

This study sought to identify pacing strategies for the five prescribed test for competitors in the men's and women's Rx divisions of the 2020 CFO. The data suggests that the chief predictors for completion rate on four of the five tests were related to prescribed calisthenic-gymnastics components. Test 1 was best predicted by average BFB completion rate, test 2 by variability in TTB rate, test 3 by average HSPU rate, and test 5 by the average number of RMU repetitions completed per set. The only instances where a gymnastic component was not the best predictor were on test 4 and test 5's tie-break time. Additional factors were also identified to produce the best combination of considerations for maximizing performance on each test. Except for body mass on test 5, the additional predictors included as part of each test's best combination were facets of pacing strategy. Neither sex-division nor any competition experience variable was found worthy of inclusion in any model. This study builds upon a pilot study of 2016 CFO pacing strategies (18) by using a much larger sample (*n* = 160 vs. *n* = 11) of higher ranking competitors (All top 10,000 vs. recreational competitors who all ranked outside the top 10,000) and identifying more specific strategies to aid athletes and coaches in their approach to each test.

The average (or the variability of) calisthenic-gymnastics repetition completion rates over the entire test duration was the best predicter of performance on four out of five tests. Those who could perform these exercise types at a more consistent and faster rate (or with less breaks) throughout each test scored better. This finding helps clarify an existing hypothesis that a faster, more consistent overall pace is best for performance (6, 18) and also agrees with and helps clarify the previously observed distinctions between sex divisions and across ranks for many of the same exercises (17). It had been thought plausible that the greater upper-body strength and endurance typically seen in men was responsible for the faster pace in gymnastics they maintained over women across all ranks (21, 22), especially when given the same workloads (2). However, that same investigation also highlighted the finding that most but not all gymnastics-type exercises favored men (i.e., top 10% women outperformed non-top 10% men in HSW) (17). With sex-division not being identified as a significant predictor on any test in this study, it may be more prudent to avoid the assumption that performance in calisthenic-gymnastics components will favor men when included in a CrossFit®-style workout or CFO test; at least, amongst higher-ranking (top 10,000) competitors. Rather, adopting a view that while men might possess a natural advantage, individual skill in such movements is the ultimate determinant. Interestingly, research has rarely considered competency in these types of movements when aiming to predict performance and should be considered in future studies. According to a recent meta-analysis (23), only one (out of 21 studies) has reported gymnastics performance to be relevant in a CrossFit®-style workout. Leitão et al. (24) noted a strong, negative relationship between maximum pull-ups and time to complete the benchmark workout “Fran”; a predictable outcome considering half of “Fran” prescription requires pull-up (45 total repetitions). While it would be impractical to perform maximal testing on every exercise appearing in CrossFit®-style workouts just for the sake of prediction, some method of assessing calisthenic-gymnastics capability or capacity seems worthy of consideration.

The strategy employed for resistance training exercises provided additional insight after calisthenic-gymnastics pacing. All five tests incorporated an exercise that required competitors to lift (tests 1–4) or project (test 5) an external load, and tests 1, 3, and 4 alternated this component with a calisthenic-gymnastic (tests 1 and 3) or plyometric-body weight (test 4) exercise on each set (2). According to regression analysis, the importance of either movement type varied across tests and appeared to be affected by the exercise's complexity and/or prescribed intensity. For example, the comparatively lower intensity loads prescribed for tests 2 and 5 (i.e., DBT and WB, respectively) had no direct influence on test completion rate. Then, G2OH rate over the last half of test 1 was influential on completion rate (*β* = 9.55) but at a fraction of BFB's influence (*β* = 36.84). However, when higher relative (to average body mass) loads were prescribed over the last half of test 3 for DL, the effect on test completion rate was nearly twice (*β* = 11.27) that of the gymnastics components prescribed over the entire test (*β* = 5.74) and −1.5× greater than last-half gymnastics (i.e., HSW) specifically (*β* = 7.56). Still, it must be understood that within the design of test 3, the ability to complete DL repetitions at a faster rate over the last half was only relevant if the competitor could complete all first-half HSPU repetitions in a timely manner (i.e., the best predictor of test 3 performance). Finally, when complex gymnastics were replaced by plyometric-body weight exercises (BJ-SLSQ) and paired with a more complex weightlifting exercise (i.e., CNJ), test performance was only be predicted by CNJ pacing strategy. These findings help clarify when indices of strength might be important. Tibana et al. demonstrated stronger relationships (*r* = 0.72–0.92) between several strength measures and 2020 CFO test 3 and 4 performance compared to those observed for aerobic and muscular endurance metrics in a small sample of 11 men and 6 women (25). Whereas, the authors noted weaker relationships between the same indices of strength and all other tests, with only Olympic lifting ability showing relationships of comparable strength. Others have also noted variability in relationships between strength measures and CFO performance (overall rank or on individual tests from other years) (12, 14, 15, 26, 27), but differences in studied variables make it difficult to determine whether those relationships follow a similar pattern. Regardless, exercise complexity and load (when applicable) appear to modulate the importance of calisthenic-gymnastics and resistance training exercises when they appeared in 2020 CFO tests.

Though sustaining the fastest possible repetition completion rate should logically produce the best score in a CrossFit®-style workout or test (6, 18), this does not suggest that an “all out” pace is always advisable or even possible. Anecdotal evidence suggests that athletes will identify “rest” opportunities within the programming to enable greater effort on other facets (28). Even if these types of workouts typically produce physiological post-exercise responses that are akin to high-intensity or “vigorous” efforts (4), it is unlikely that the same intensity of effort is given over their entirety. Changes in body position and level, as well as between-exercise transitions, would have simultaneously and naturally disrupted pace and physiological steady state, and elevated oxygen and energy demands (29–31). At 9–20 min, the duration of the five tests examined in this study would have simply been too long to sustain an “all out” pace. Indeed, previous analysis revealed several differences in repetition completion rate employed for each exercise, as well as in how they changed over course of each test (17). While many between-exercise differences are naturally-occurring (e.g., DU repetitions are naturally faster than ROW and WB), it is unknown whether the differences noted between the first and last half were purposeful, due to fatigue, or a function of prescription changes (e.g., the prescription for tests 3 and 4 were different between halves). It is possible, however, that through the exclusion of certain variables from the final models, stepwise regression provided an indication about which facets were commonly used by competitors to “rest”. Ignoring failed repetitions because they can be assumed to have been unintentional, stepwise regression excluded all pacing strategy aspects about the following exercises in their respective test's final models: G2OH (test 1, first half only), DBT (test 2), DL (test 3, first half only), exercise transitions (test 3 and 4), BJ-SLSQ (test 4), WB (test 5 rate and tie-break time), and ROW (test 5). Since performance on these still contributed to their respective test's final score, their exclusion from the best combinations might suggest greater freedom in execution.

Another study expectation was that past CFO experiences would affect the optimal pacing strategy. Contrary to past evidence (16), competition experience was not as important for predicting test completion rate when considered alongside pacing strategy. Though previous CFO ranks (ever and in 2019) individually explained 23%–61% of variance on each test, neither were included in a final model for any test, even after ignoring study parameters (i.e., 8-variable limit, validation-cross validation model consistency, normality of residuals). Meanwhile, CFO participation (i.e., consecutive and skipped) only became relevant after considering several other variables outside of study parameters (i.e., after 10 variables on test 3 and after five variables on test 4 but not in each validation groups). This occurred despite greater correlation coefficients in this study for past CFO ranks than what had been previously reported for the same five CFO tests (16). The reasons for why experience was not more influential could be related to sample quality and differences in study aims. Mangine and McDougle (16) only looked at men who ranked within the top 1,000 of the 2020 competition and were not attempting to determine the most influential competitive experiences on performance. Approximately 32% of that sample had advanced beyond the CFO at least once, and those competitors may have placed less importance on earning the highest CFO rank possible in favor of simply performing well enough to advance. Amongst the men and women who have won the CFO, only 10 (−7.1%) have also won the Games™, six have ranked outside of the top 10, and three failed to qualify. Conversely, Games™ winners have ranked within the top 20 CFO competitors on 24 out of 26 possible occasions (i.e., 13 possible occasions for men and women each) (19). Thus, although the present data suggest that past CFO experiences are more important for the top 10,000 competitors than the top 1,000, that experience was less important than pacing strategy for 2020 CFO performance.

The influential pacing tactics identified for 2020 CFO tests should be viewed within the context of this study's limitations. The CFO rules provide competitors with the option of completing tests in front of a certified judge at a CrossFit® affiliate or submitting a recording of their effort to be judged by competition officials (2, 3). Several reasons are possible for why these athletes chose to submit videos for all five CFO tests while others did not. None can be confirmed but might include not having access to a certified judge or CrossFit® affiliate on one or more tests. One might argue that not having such access could be indicative of a sub-group of athletes who do not regularly train at a CrossFit® affiliate. Or, given that all competitors ranked within the top 10,000 and produced highly competitive scores on each test, it is likely that each possessed documentation (i.e., a video recording) that would have been made available upon request. Relatedly, the decision to only include athletes who submitted scores for all five tests and ranked within the top 10,000 helped ensure our sample included high-ranking but well-rounded CrossFit® athletes, and not specialists (i.e., those who only submit scores for tests that best fit their personal skillsets). The drawback to this requirement implies that better pacing strategies might exist, though they may only be relevant to the most elite competitors and/or those with very specific physiological traits. Another uncertainty about the observed strategies was the specific set of conditions under which each video-recorded attempt was made. Competitors were free to attempt each test as often as needed so long as they submitted their score when they were due (2, 3). The submissions reviewed in this study could have been of an athlete's first and only attempt on one or more tests, an optimal strategy determined from several submaximal, practice trials, or even one of several maximal attempts made within the 4-day submission period. It cannot be known whether practice, accumulated fatigue, and/or an athlete's health status (i.e., whether they were injury free) influenced our observations. That said, the randomized case selection process should have limited their potential influence to acceptable levels of random chance (i.e., *α* = 0.05). Likewise, random case selection should have also minimized the variability seen in each competitor's ability to satisfy pre-defined technical standards and produce a valid score, an effect also minimized by the decision to only examine video submissions that had already been certified by competition officials (3) and subsequently our research team. Still, our research team was only capable of identifying clear and major infractions (e.g., miscounted repetitions and incomplete efforts) for removal. It is possible for a variety of minor infractions (e.g., partial repetitions) to have occurred and affected our observations, but because these could not be verified with 100% certainty, they were retained for analysis.

In conclusion, the two most common scoring structures for CFO tests ask competitors to complete as many repetitions as possible within a set time limit or to complete a stated number of repetitions in the least amount of time. Within a single competition, each CFO test is unique in design and is unknown to competitors prior to when competition officials reveal its details and give allow only a few days for them to produce their best effort (2, 3). Athletes must reconcile their personal set of skills and physiological traits with a correct assessment of a test's fitness requirements to best devise their approach. The present study determined that across each of the 2020 CFO tests, athletes who performed calisthenic-gymnastics movements at a faster and more consistent rate scored better. Athletes should primarily aim their focus on identifying a strategy (e.g., repetitions per set, break count and duration) that maximizes their ability to complete required repetitions for these exercise types at the fastest and most consistent rate possible. Or, in the case of tie-break structure for test 5, those who significantly lacked gymnastics skill did best when they minimized the time performing, and transitioning to, this exercise type.

A secondary finding suggested that the attention placed on strategizing calisthenics-gymnastics movements might be diverted to performance on resistance training exercises, particularly when higher external loads and/or more complex exercises were programmed. When lower intensity/complexity resistance training exercises were paired with low-complexity calisthenic-gymnastics, the latter component was more important. However, when intensity load and/or complexity was greater for the resistance training exercise, it became more influential, but that relevance was still subject to programming (e.g., when the exercise became relatively heavy in the test). It is also worth noting that when complex gymnastics or higher intensity/complexity resistance training exercises were present in a test, performance in these movements often affected the relevance of remaining exercises and even transition (between exercises) strategy. In such scenarios, athletes might view the less relevant components as opportunities to “rest” (i.e., perform at a relatively less aggressive rate) to preserve energy and limit the accumulation of fatigue.

Although experience and familiarity with similarly-structured CrossFit®-style workouts and CFO tests might help with recognizing an appropriate strategy, the present data did not find experience to be relevant for competitors who already ranked within the top 10,000. Likewise, the combination of known physiological and programming differences between sexes were hypothesized as potential moderators of strategy but did not alter the most predictive pacing tactics for each test. The identified strategies appear to be useful for competitors within both sex-divisions. Since these strategies may be limited to the five tests of the 2020 CFO, future endeavors will undoubtedly wish to confirm these results in other collections of CrossFit®-style workouts and CFO tests. Before doing so, a great deal of time and effort might be saved by investigating methods of classifying workouts. With an infinite number of possible programming combinations, it may be more efficient to determine optimal pacing strategies for specific classification or “types” instead of the specific workouts themselves. Any resultant recommendations and/or discovered relationships with measured physiological traits will then become more generalizable. Currently, however, no scientific and objective method for classifying CrossFit®-style and high-intensity functional training workouts exists.

## Data Availability

Publicly available datasets were analyzed in this study. This data can be found here: https://games.crossfit.com/Leaderboard/open/.

## References

[1] Glassman G. *Crossfit training guide level 1: The CrossFit Journal*. (2011).

[2] CrossFit. *Open* *Workouts. CrossFit Games*. (2021). Available at: https://games.crossfit.com/workouts/open/2021 (accessed August 16, 2022).

[3] CrossFit. *Games competition rulebook: The CrossFit Journal*. (2022).

[4] McDougle JM Mangine GT Townsend JR Jajtner AR Feito Y. Acute physiological outcomes of high-intensity functional training: a scoping review. PeerJ. (2023) 11:e14493. 10.7717/peerj.14493 36620744 PMC9817969

[5] CrossFit. *Finding the Fittest on Earth. CrossFit Games*. (2022). Available at: https://games.crossfit.com/history-of-the-games (accessed August 16, 2022).

[6] Mangine GT Seay TR. Quantifying CrossFit®: potential solutions for monitoring multimodal workloads and identifying training targets. Front SportsAct Liv. (2022) 4:949429. 10.3389/fspor.2022.949429 PMC9613943 36311217

[7] Skorski S Mujika I Bosquet L Meeusen R Coutts AJ Meyer T. The temporal relationship between exercise, recovery processes, and changes in performance. Int J Sports Physiol Perform. (2019) 14(8):1015–21. 10.1123/ijspp.2018-0668 31172832

[8] Butcher SJ Neyedly TJ Horvey KJ Benko CR. Do physiological measures predict selected CrossFit® benchmark performance? Open Access J Sports Med. (2015) 6:241. 10.2147/OAJSM.S88265 26261428 PMC4527742

[9] Bellar D Hatchett A Judge L Breaux M Marcus L. The relationship of aerobic capacity, anaerobic peak power and experience to performance in CrossFit exercise. Biol Sport. (2015) 32(4):315–20. 10.5604/20831862.1174771 26681834 PMC4672163

[10] Feito Y Burrows EK Tabb LP. A 4-year analysis of the incidence of injuries among CrossFit-trained participants. Orthop J Sports Med. (2018) 6(10):2325967118803100. 10.1177/2325967118803100 30370310 PMC6201188

[11] Dexheimer JD Schroeder ET Sawyer BJ Pettitt RW Aguinaldo AL Torrence WA. Physiological performance measures as indicators of CrossFit® performance. Sports. (2019) 7(4):93. 10.3390/sports7040093 31013585 PMC6524377

[12] Zeitz EK Cook LF Dexheimer JD Lemez S Leyva WD Terbio IY The relationship between crossfit® performance and laboratory-based measurements of fitness. Sports. (2020) 8(8):112. 10.3390/sports8080112 32796573 PMC7466681

[13] Carreker JDD Grosicki GJ. Physiological predictors of performance on the CrossFit® “murph” challenge. Sports. (2020) 8(7):92. 10.3390/sports8070092 32605265 PMC7404702

[14] Mangine GT Tankersley JE McDougle JM Velazquez N Roberts MD Esmat TA Predictors of CrossFit open performance. Sports. (2020) 8(7):102. 10.3390/sports8070102 32698335 PMC7404807

[15] Martínez-Gómez R Valenzuela PL Barranco-Gil D Moral-González S García-González A Lucia A. Full-squat as a determinant of performance in CrossFit. Int J Sports Med. (2019) 40(09):592–6. 10.1055/a-0960-9717 31291652

[16] Mangine GT McDougle JM. Crossfit® open performance is affected by the nature of past competition experiences. BMC Sports Sci Med Rehabil. (2022) 14(1):1–15. 10.1186/s13102-022-00434-0 35331301 PMC8944014

[17] Mangine GT Zeitz EK Dexheimer JD Hines A Lively B Kliszczewicz BM. Pacing strategies differ by sex and rank in 2020 CrossFit®open tests. Sports. (2023) 11(10):199. 10.3390/sports11100199 37888526 PMC10611042

[18] Mangine GT Feito Y Tankersley JE McDougle JM Kliszczewicz BM. Workout pacing predictors of crossfit open performance: a pilot study. J Hum Kinet. (2021) 78(1):89–100. 10.2478/hukin-2021-0043 34025867 PMC8120962

[19] Leaderboard. *Leaderboard 2021*. Available at: http://games.crossfit.com/leaderboard (accessed August 16, 2022).

[20] Weir JP Vincent WJ. Multiple correlation and multiple regression. Statistics in Kinesiology. Champaign, IL. 5th ed. Human Kinetics (2020). p. 103–14.

[21] Sandbakk Ø Solli GS Holmberg H-C. Sex differences in world-record performance: the influence of sport discipline and competition duration. Int J Sports Physiol Perform. (2018) 13(1):2–8. 10.1123/ijspp.2017-0196 28488921

[22] Hunter SK. The relevance of sex differences in performance fatigability. Med Sci Sports Exerc. (2016) 48(11):2247. 10.1249/MSS.0000000000000928 27015385 PMC5349856

[23] Meier N Schlie J Schmidt A. Crossfit®: “unknowable” or predictable?—a systematic review on predictors of CrossFit® performance. Sports. (2023) 11(6):112. 10.3390/sports11060112 37368562 PMC10304543

[24] Leitão L Dias M Campos Y Vieira JG Sant'Ana L Telles LG Physical and physiological predictors of FRAN CrossFit(®) WOD athlete’s performance. Int J Environ Res Public Health. (2021) 18(8):4070. 10.3390/ijerph18084070 33921538 PMC8069540

[25] Tibana RA de Sousa Neto IV Sousa N Romeiro C Hanai A Brandão H Local muscle endurance and strength had strong relationship with CrossFit® open 2020 in amateur athletes. Sports. (2021) 9(7):98. 10.3390/sports9070098 34357932 PMC8309786

[26] Martínez-Gómez R Valenzuela PL Alejo LB Gil-Cabrera J Montalvo-Pérez A Talavera E Physiological predictors of competition performance in CrossFit athletes. Int J Environ Res Public Health. (2020) 17(10):3699. 10.3390/ijerph17103699 32456306 PMC7277742

[27] Schlegel P Režný L Fialová D. Pilot study: performance-ranking relationship analysis in Czech crossfiters. J Hum Sport Exerc. (2021) 16(1):187–98. 10.14198/jhse.2021.161.17

[28] Nief T. *CrossFit Pacing: The Key To Perfectly Executing Workouts Online: WODprep*. (2019). Available at: https://wodprep.com/blog/crossfit-pacing-workouts/ (accessed August 16, 2022).

[29] Herda T Cramer J. Bioenergetics of exercise and training. In: Haff G Triplett N, editors. Essentials of Strength Training and Conditioning. Champaign, IL: Human Kinetics (2016). p. 43–64.

[30] Reuter B Dawes JJ. Program design and technique for aerobic endurance training. In: Haff G Triplett N, editors. Essentials of Strength Training and Conditioning. Champaign, IL: Human Kinetics (2016). p. 559–82.

[31] Kliszczewicz B Snarr RL Esco MR. Metabolic and cardiovascular response to the CrossFit workout “Cindy”. J Sport Hum Perform. (2014) 2(2):1–9. 10.12922/jshp.0038.2014

